# Insulin and glucose metabolism with olanzapine and a combination of olanzapine and samidorphan: exploratory phase 1 results in healthy volunteers

**DOI:** 10.1038/s41386-021-01244-7

**Published:** 2021-12-09

**Authors:** Frederico G. S. Toledo, William F. Martin, Linda Morrow, Carine Beysen, Daiva Bajorunas, Ying Jiang, Bernard L. Silverman, David McDonnell, Mark N. Namchuk, John W. Newcomer, Christine Graham

**Affiliations:** 1 Division of Endocrinology and Metabolism, Department of Medicine, University of Pittsburgh, Pittsburgh, PA USA; 2 Alkermes, Inc., Waltham, MA USA; 3 ProSciento, Inc., Chula Vista, CA USA; 4 Vault Bioventures, San Diego, CA USA; 5 Alkermes Pharma Ireland Limited, Dublin, Ireland; 6 Thriving Mind South Florida, Miami, FL USA; 7 Washington University School of Medicine, St. Louis, MO USA; 8 Present Address: DBMD Consulting, Pompano Beach, FL USA; 9 Present Address: Department of Biological Chemistry and Molecular Pharmacology, Blavatnik Institute, Harvard Medical School, Boston, MA USA

**Keywords:** Medical research, Developmental biology

## Abstract

A combination of olanzapine and samidorphan (OLZ/SAM) received US Food and Drug Administration approval in May 2021 for the treatment of adults with schizophrenia or bipolar I disorder. OLZ/SAM provides the efficacy of olanzapine, while mitigating olanzapine-associated weight gain. This exploratory study characterized the metabolic profile of OLZ/SAM in healthy volunteers to gain mechanistic insights. Volunteers received once-daily oral 10 mg/10 mg OLZ/SAM, 10 mg olanzapine, or placebo for 21 days. Assessments included insulin sensitivity during an oral glucose tolerance test (OGTT), hyperinsulinemic-euglycemic clamp, other measures of glucose/lipid metabolism, and adverse event (AE) monitoring. Treatment effects were estimated with analysis of covariance. In total, 60 subjects were randomized (double-blind; placebo, *n* = 12; olanzapine, *n* = 24; OLZ/SAM, *n* = 24). Olanzapine resulted in hyperinsulinemia and reduced insulin sensitivity during an OGTT at day 19, changes not observed with OLZ/SAM or placebo. Insulin sensitivity, measured by hyperinsulinemic-euglycemic clamp, was decreased in all treatment groups relative to baseline, but this effect was greatest with olanzapine and OLZ/SAM. Although postprandial (OGTT) glucose and fasting cholesterol concentrations were similarly increased with olanzapine or OLZ/SAM, other early metabolic effects were distinct, including post-OGTT C-peptide concentrations and aspects of energy metabolism. Forty-nine subjects (81.7%) experienced at least 1 AE, most mild or moderate in severity. OLZ/SAM appeared to mitigate some of olanzapine’s unfavorable postprandial metabolic effects (e.g., hyperinsulinemia, elevated C-peptide) in this exploratory study. These findings supplement the body of evidence from completed or ongoing OLZ/SAM clinical trials supporting its role in the treatment of schizophrenia and bipolar I disorder.

## Introduction

Antipsychotic pharmacotherapy is the cornerstone of treatment for schizophrenia, but this class of medications has been associated with the development of metabolic sequelae, including increased risk of type 2 diabetes [1, 2]. A large population cohort study from Denmark found that the risk of developing diabetes was more than threefold higher in individuals with schizophrenia compared with those without schizophrenia. In addition, incident diabetes risk was increased more than threefold with antipsychotic treatment vs. no treatment, and the risk of diabetes development was similar between first-generation antipsychotics (adjusted hazard ratio, 3.06; 95% confidence interval [CI], 1.32–7.05) and second-generation antipsychotics (adjusted hazard ratio, 3.44; 95% CI, 1.73–6.83) when compared among patients who were antipsychotic naive, and after adjustment for potential confounders, including a family history of diabetes [3].

Of the available antipsychotic treatments used for schizophrenia, the risk of incident diabetes and dyslipidemia is higher with olanzapine than with other agents [4–6]. This, along with the risk of substantial weight gain [1, 7, 8], has limited olanzapine’s overall clinical utility [9, 10]. Although the significant weight gain associated with olanzapine is likely to impose metabolic perturbations over the long term, studies in healthy volunteers and patients with schizophrenia have revealed that changes in glucose and lipid metabolism can occur quickly, as early as 3 days after starting olanzapine treatment [11–14]. Therefore, apart from the long-term metabolic impact of weight gain, the initial metabolic changes associated with olanzapine likely reflect weight-independent pharmacodynamic effects of the drug.

A combination of olanzapine and samidorphan (OLZ/SAM) has been approved by the US Food and Drug Administration for the treatment of adults with schizophrenia or bipolar I disorder [15]. Olanzapine is among the most effective antipsychotic agents available [1, 8]. Samidorphan, a new molecular entity, binds in vitro with high affinity to human µ-, κ-, and δ-opioid receptors, and acts as an antagonist at μ-opioid receptors and as a partial agonist at κ- and δ-opioid receptors [16, 17]. In vivo, samidorphan functions as an opioid receptor antagonist [18]. The addition of samidorphan mitigates olanzapine-associated weight gain without compromising the established antipsychotic efficacy of olanzapine [19–21].

Preclinically, samidorphan reduced the rate of weight gain and mitigated some metabolic abnormalities when co-administered with olanzapine [22]. In clinical studies, the weight-mitigating effects of samidorphan are observed as early as 4–6 weeks after initiating treatment [19, 23]. However, whether the two treatments differentiate on early metabolic effects is unknown. This 3-week, exploratory, mechanistic study sought to characterize short-term effects of OLZ/SAM relative to olanzapine on insulin sensitivity and additional metabolic parameters, including endogenous glucose production, lipid metabolism, energy intake, and energy expenditure, among others. Healthy volunteers were enrolled to avoid metabolic confounders, such as weight, metabolic irregularities, smoking, and/or prior exposure to antipsychotics or mood stabilizers, which may be present in patients with schizophrenia [2, 24, 25]. The use of healthy volunteers negated the predisposition to metabolic derangements associated with severe mental illness and precluded the need to monitor schizophrenia symptom status during the study. Furthermore, concern that the multiple, intensive study procedures may have been perceived as too intrusive for patients with schizophrenia provided further support for conducting the study in healthy volunteers. Finally, healthy volunteers were enrolled owing to the exploratory and hypothesis-generating nature of the study, with the intention of providing a guide for future studies in patients with schizophrenia.

## Methods

This phase 1, randomized, double-blind, parallel-group, single-center study in healthy volunteers (NCT02922426) was conducted from September 2016 to July 2017 in accordance with the ethical principles of the Declaration of Helsinki and with the International Council for Harmonisation Good Clinical Practice guidelines. Study materials and the protocol were reviewed and approved by an institutional review board/independent ethics committee. All participants provided written, informed consent before initiation of any study-specific procedures. The samidorphan dose used in this study (10 mg in OLZ/SAM) was previously identified as the optimal dose for efficacy, tolerability, and weight gain mitigation in patients with schizophrenia receiving OLZ/SAM [19]. A 10 mg dose of olanzapine was utilized in OLZ/SAM and as monotherapy, because it is the maximum tolerated dose for repeat administrations in healthy subjects.

### Study design

**Fig. 1:**
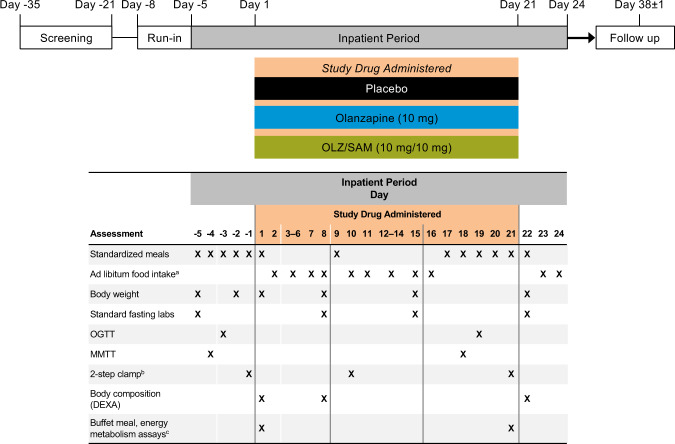
Study design schematic. After screening and a run-in period, subjects were admitted to the clinic on day −5 and were subsequently randomized on day 1 to placebo, olanzapine, or OLZ/SAM for 21 days. The study assessed a variety of prespecified endpoints related to glucose, lipid, and energy metabolism. Assessments taken during the inpatient period are depicted in the table. Prior to days when metabolic tests (i.e., OGTT, MMTT, clamp, or indirect calorimetry procedures) were scheduled, subjects received a standardized dinner and were required to fast for 10 h prior to the procedure. DEXA, dual energy x-ray absorption; EGP, endogenous glucose production; MMTT, mixed-meal tolerance test; OGTT, oral glucose tolerance test; OLZ/SAM, combination of olanzapine and samidorphan. ^a^Subjects could choose meals (30% to 45% fat content) or other snacks. ^b^Two-step hyperinsulinemic-euglycemic clamp assessed by infusion of stable isotope-labeled glucose. Labeled glucose was also administered prior to the clamp procedure to determine fasting endogenous glucose production. ^c^Assessments of energy metabolism included resting metabolic rate, respiratory quotient, and substrate oxidation.

This 3-week study included a screening visit, 3-day run-in period, inpatient period, and follow-up visit (Fig. 1). During the in-house run-in period, no active treatments were given, but subjects wore an activity monitor and recorded their food consumption in a diary. Subjects remained at the study site for ~29 days, starting on day −5. On day 1, subjects were randomized 2 : 2 : 1 in a double-blind manner using a single master randomization schedule generated by an independent vendor (ProSciento, Inc., Chula Vista, CA) to once-daily, oral OLZ/SAM, olanzapine, or placebo for 21 days, during which time study investigators and subjects were blinded to treatment. A follow-up safety assessment occurred ~2 weeks after subjects left the study site. Metabolic and body composition assessments were conducted before, during, and at the end of the treatment period (Fig. 1). There was no formal sample size calculation, as the study was exploratory in nature.

### Study population

Eligible subjects were adults 18–40 years of age, with a body mass index of 18 to <25 kg/m^2^ at screening and randomization. Subjects had stable body weight that had not changed more than 4% in the preceding 2 months. Subjects were excluded for the presence of a clinically significant medical or psychiatric condition, laboratory abnormality, or history of suicidal ideation or behavior (as assessed by the Columbia-Suicide Severity Rating Scale [C-SSRS] [26] at screening and clinic admission). In addition, a history of diabetes, hemoglobin A1c (HbA1c) levels >5.7% or low neutrophil count (≤1.5 × 10^3^/μL) at screening, engagement in regular structured exercise (>30 min daily on ≥3 days/week), dependence on any substance other than caffeine [27], or history of regular smoking or nicotine use were exclusionary. Subjects were also excluded based on known or suspected intolerance, allergy, or hypersensitivity to olanzapine or opioid antagonists, current or anticipated use of prescribed opioids (e.g., for a planned surgery), or positive urine screen for amphetamines, barbiturates, cannabinoids, or cocaine.

### Assessments

Although the study was exploratory in nature, a variety of pharmacodynamic assessments were prespecified for the purpose of measuring effects on carbohydrate and lipid metabolism, energy expenditure, and food intake (see details in Supplementary Materials). A schematic of the clamp procedure is provided in Supplementary Fig. S1.

### Statistical analysis

The primary objective of this study was to characterize insulin sensitivity, as well as changes in carbohydrate and lipid metabolism, following treatment with OLZ/SAM and olanzapine. Because of the exploratory nature of the study, there were no prespecified primary or key secondary outcomes and no control for multiplicity. Safety was assessed from all randomized subjects who received at least 1 dose of study drug; pharmacodynamic assessments were conducted in subjects who received at least 1 dose of study drug and who adequately complied with the protocol (no deviations related to investigational product intake) as determined by the sponsor and had sufficient data for at least 1 pharmacodynamic or body composition endpoint. Analysis of covariance (ANCOVA) was used to assess treatment effects on metabolic endpoints. There was a higher proportion of black or African American subjects in the OLZ/SAM and placebo groups vs. the olanzapine group after randomization. Consequently, adjustments were made to the regression model to correct for the race imbalance. Specifically, the ANCOVA model included treatment as a factor and the baseline of the dependent variable and race (black/African American or not) as covariates. Results are presented as least square (LS) means or geometric LS mean (GLSM) ratios with 90% CIs. The analysis of pharmacodynamic endpoints was based on observed results, without imputation for missing data. Safety variables, including weight, were summarized using descriptive statistics. Reported adverse events (AEs) were coded using the Medical Dictionary for Regulatory Activities v19.1 based on preferred terms and system organ class categories.

## Results

### Subject disposition

Of 130 subjects screened, 60 met eligibility criteria and were randomized, 12 to the placebo group, and 24 each to the olanzapine and OLZ/SAM groups (Supplementary Fig. S2). All 60 subjects were included in assessments of pharmacodynamics and safety. In total, 52 (86.7%) subjects completed the study: 11 (91.7%) in the placebo group, 22 (91.7%) in the olanzapine group, and 19 (79.2%) in the OLZ/SAM group. Reasons for early discontinuation were AEs (*n* = 6 overall [10.0%]; 2 from the olanzapine group and 4 from the OLZ/SAM group) or withdrawal by the subject (*n* = 2 overall [3.3%]; 1 each from the placebo and OLZ/SAM groups).

### Baseline characteristics

Most subjects were male (81.7%) and the overall mean (SD) age was 28.4 (5.4) years. The three treatment groups were balanced at baseline for sex, age, weight, body mass index, percentage body fat, and HbA1c levels (Table 1). Despite randomization, more White subjects were included in the olanzapine group.

**Table 1:** Demographics and subject characteristics at screening: pharmacodynamics population.

Parameter	Placebo (*n* = 12)	Olanzapine (*n* = 24)	OLZ/SAM (*n* = 24)
Male, *n* (%)	10 (83.3)	20 (83.3)	19 (79.2)
Age, mean (SD), years	30.0 (7.2)	28.0 (4.7)	28.0 (5.0)
Race, *n* (%)			
White	7 (58.3)	20 (83.3)	10 (41.7)
Black or African American	5 (41.7)	2 (8.3)	13 (54.2)
American Indian/Alaska native	0	0	1 (4.2)
Asian	0	1 (4.2)	0
Multiple races	0	1 (4.2)	0
Weight, mean (SD), kg	69.6 (7.0)	70.7 (7.5)	71.4 (7.7)
Body mass index, mean (SD), kg/m^2^	23.0 (1.9)	23.2 (1.3)	23.5 (1.2)
Percentage body fat, mean (SD)	23.5 (10.8)	24.7 (8.6)	22.6 (10.8)
Fat-free mass,^a^ mean (SD), kg	53.5 (9.0)	53.4 (8.8)	55.3 (9.7)
HbA1c, mean (SD), %	5.2 (0.3)	5.4 (0.2)	5.3 (0.2)

*HbA1c* hemoglobin A1c, *OLZ/SAM* combination of olanzapine and samidorphan.

^a^Fat-free mass was measured at the baseline visit.

### Pharmacodynamic parameters

#### Fasting glucose, insulin, and homeostatic model of insulin resistance

Fasting glucose concentrations were similar at baseline across the three treatment groups, with similar decreases across all groups from baseline to day 22. For fasting insulin, a small decrease was observed for placebo compared with small similar increases observed with both olanzapine and OLZ/SAM (Supplementary Table S1).

Small changes in the homeostatic model of insulin resistance were observed in all treatment groups from baseline to day 22, with olanzapine and OLZ/SAM both resulting in small numeric increases compared with a decrease observed with placebo (Supplementary Table S1).

#### Fasting lipids and adipokines

Fasting total cholesterol and low-density lipoprotein cholesterol increased similarly with olanzapine and OLZ/SAM from baseline to day 22; the increases were larger than the changes observed with placebo. Triglycerides also increased for olanzapine, but increases were minimal and similar for OLZ/SAM and placebo treatment. high-density lipoprotein cholesterol decreased comparably across all treatment groups from baseline to day 22 (Supplementary Table S1). Leptin increased similarly for both olanzapine and OLZ/SAM (LS mean change [90% CI]: 2.09 [0.52, 3.67] and 2.31 [0.93, 3.69], respectively) and the increases were larger than that observed for placebo (0.33 [−1.73, 2.38]).

#### Plasma concentrations of glucose, insulin, and C-peptide during tolerance tests

**Fig. 2:**
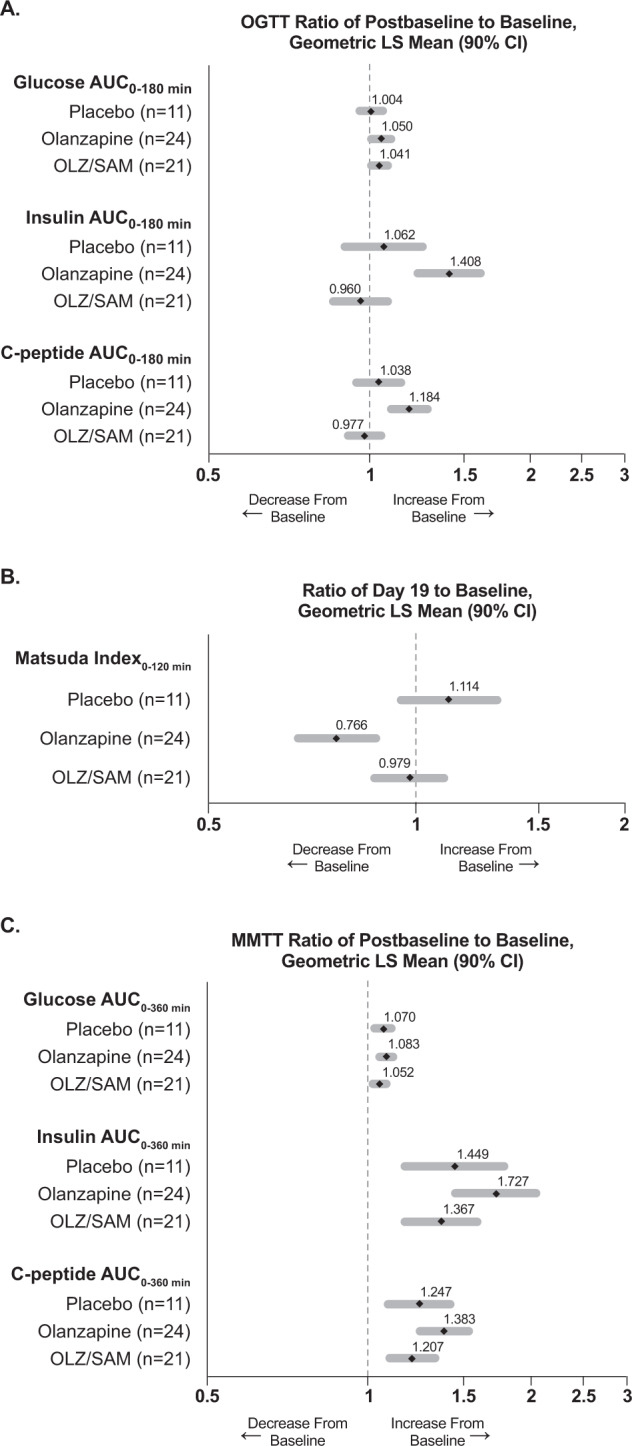
Ratios of glucose, insulin, and C-peptide concentrations and changes in the Matsuda Index. The effects of olanzapine and OLZ/SAM on postprandial glucose-related parameters were assessed after oral administration of 75 g of glucose for 180 min (OGTT) or (2) 8-ounce bottles of Ensure Plus for 360 min (MMTT). The ratio of change in glucose, insulin, and C-peptide values at day 19 relative to baseline for the OGTT (Fig. 2A) and at day 18 relative to baseline for the MMTT (Fig. 2C) were determined using ANCOVA models, with treatment as a factor and baseline of the dependent variable and race (black/African American or not) as covariates. Changes in insulin sensitivity at day 19, calculated as the Matsuda index from OGTT results, are shown by treatment group (Fig. 2B). Decreases in the Matsuda index indicate a decline in insulin sensitivity. ANCOVA, analysis of covariance; AUC_0-180_, area under the plasma concentration-time curve from time 0 to 180 min; AUC_0-360_, area under the plasma concentration-time curve from time 0 to 360 min; CI, confidence interval; LS, least squares; MMTT, mixed-meal tolerance test; OGTT, oral glucose tolerance test; OLZ/SAM, combination of olanzapine and samidorphan.

Glucose, insulin, and C-peptide concentrations were measured before and at intervals following an oral glucose tolerance test (OGTT) (Supplementary Fig. S3A). At baseline, the area under the concentration vs. time curve for glucose (AUC_Glu_) was similar across all treatment groups (mean [SD] values of 442.07 [75.01], 425.21 [70.98], and 396.47 [48.14] h*mg/dL, respectively, for placebo, olanzapine, and OLZ/SAM). When the OGTT was repeated at day 19, there were small changes in all groups from baseline in AUC_Glu_ (mean change [SD] of −6.29 [61.07], 15.92 [39.09], and 22.57 [50.30] h*mg/dL, respectively, for placebo, olanzapine, and OLZ/SAM). Figure 2A depicts relative changes in AUC for glucose, insulin, and C-peptide at day 19 vs. baseline.

Larger group differences were observed for changes in AUC_insulin_ from baseline to day 19 (Fig. 2A). At baseline, AUC_insulin_ was generally similar across the three treatment groups (mean [SD] values of 282.73 [204.47], 223.71 [130.88], and 208.13 [163.36] h*µU/mL, respectively, for placebo, olanzapine, and OLZ/SAM). When the OGTT was repeated on day 19, there was an increase from baseline in AUC_insulin_ for olanzapine (mean [SD] change of 91.64 [94.35] h*µU/mL) that was not observed with OLZ/SAM or placebo (mean [SD] changes of −17.46 [100.73] and −2.96 [80.60] h*µU/mL, respectively; Supplementary Fig. S3A). Relative changes in AUC_insulin_ for day 19 compared with baseline are shown in Fig. 2A. Consistent with the increases in insulin observed with olanzapine treatment, greater increases were noted in C-peptide from baseline to day 19 for olanzapine.

#### Insulin secretion and insulin sensitivity during the OGTT

Glucose and insulin concentrations during the OGTT were used to estimate insulin sensitivity by the Matsuda index. At baseline, the Matsuda index was similar across the three treatment groups (mean [SD] of 3.71 [1.61], 3.91 [2.09], and 4.63 [1.95] for placebo, olanzapine, and OLZ/SAM groups, respectively). Based on the Matsuda index, there was a numeric increase from baseline at day 19 for placebo (mean change [SD] of 0.46 [1.06]) compared with a decrease observed with olanzapine (mean [SD] of −0.59 [1.44]). A smaller decrease was observed with OLZ/SAM (−0.13 [2.40]). The GLSM ratio [90% CI] of the Matsuda index at day 19 compared with baseline for all treatment groups is shown in Fig. 2B.

Olanzapine treatment resulted in an increase in the insulinogenic index (Δ0–30 min insulin/Δ0–30 min glucose) at day 19 compared with baseline (GLSM ratio [90% CI] of 1.46 [1.20, 1.77]). No significant increases were observed for placebo or OLZ/SAM between baseline and day 19 (GLSM ratios of 1.12 [0.94, 1.32] and 1.14 [0.90, 1.44], respectively).

#### Mixed-meal tolerance test

AUC profiles during the mixed-meal tolerance test (MMTT) are depicted in Supplementary Fig. S3B; the ratio of glucose, insulin, and C-peptide concentrations at day 18 relative to baseline are presented in Fig. 2C. Overall, small, similar increases in post-challenge AUC_glu_ were observed for all treatment groups at day 18 relative to baseline. Increases in post-challenge insulin and C-peptide (AUC_0–300_) were also observed in all groups from baseline to day 18. However, in line with findings from the OGTT, increases for olanzapine were numerically greater than those observed with OLZ/SAM and placebo.

#### Hepatic insulin resistance index

In an additional analysis, hepatic insulin sensitivity in the fasting state (also referred to as the hepatic insulin resistance [HIR] index) was assessed at baseline, day 10, and day 21. At baseline, values for HIR [28] corrected for fat-free mass were generally similar across treatment groups (mean [SD] of 12.93 [6.17], 16.20 [8.33], and 13.87 [6.99] nIU/L/min, respectively, for placebo, olanzapine, and OLZ/SAM). At day 10, compared with baseline, there was a small, nonsignificant increase in the HIR index in the placebo group (LS mean [90% CI] change of 1.47 [−1.93, 4.87] nIU/L/min), with larger, significant increases from baseline observed with olanzapine and OLZ/SAM (4.38 [1.55, 7.22] and 3.14 [0.64, 5.64] nIU/L/min, respectively). At day 21, the HIR index remained elevated for olanzapine (LS mean change [90% CI] of 4.89 [2.30, 7.47] nIU/L/min) but had returned to baseline for OLZ/SAM and placebo (LS mean changes of −0.52 [−2.79, 1.75] and −0.83 [−3.83, 2.17] nIU/L/min, respectively; Supplementary Fig. S4).

#### Hyperinsulinemic-euglycemic clamp

**Fig. 3:**
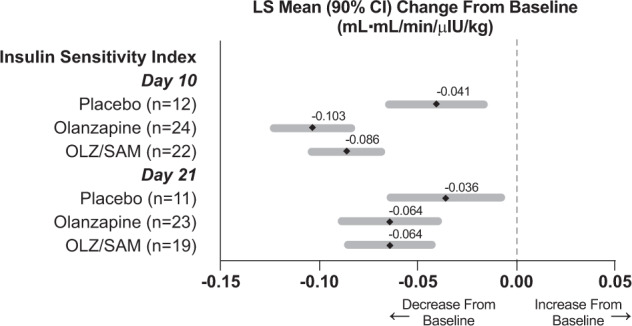
Insulin Sensitivity Index (Si) derived from hyperinsulinemic-euglycemic clamp measurements at day 10 and day 21 of treatment. Insulin sensitivity decreased with olanzapine, especially at day 10 but also at day 21. OLZ/SAM treatment produced similar changes. LS means were generated using ANCOVA, with treatment as a factor and baseline for the dependent variable and race (black/African American or not) as covariates. ANCOVA, analysis of covariance; CI, confidence interval; LS, least squares; M, glucose disposal rate based on the glucose infused per minute, corrected for fat-free mass; OLZ/SAM, combination of olanzapine and samidorphan.

To assess insulin sensitivity during the clamp procedure, a composite insulin sensitivity index (Si) was derived based on glucose infusion rates during both clamp steps (i.e., Step 1 and Step 2; Fig. 3 and Supplementary Table S2). At baseline, Si (corrected for fat-free mass) was similar across all treatment groups. At day 10, Si decreased, indicating a worsening peripheral insulin sensitivity, for all treatment groups. Decreases in Si were similar for olanzapine and OLZ/SAM, and were larger than those observed with placebo. At day 21, Si continued to be decreased from baseline in all treatment groups but to a lesser extent than at day 10. Decreases in Si at day 21 continued to be similar for olanzapine and OLZ/SAM, and larger than that observed with placebo.

#### Body weight and composition

Similar increases in body weight from baseline to day 22 were observed for olanzapine and OLZ/SAM (LS mean change [90% CI] of 2.87 [2.09, 3.65] kg and 3.16 [2.47, 3.84] kg, respectively); these increases were larger than that found with placebo (LS mean change [90% CI] of 0.57 [−0.44, 1.58] kg). Increases in fat mass were observed in all groups, with the largest increases occurring in subjects treated with olanzapine or OLZ/SAM (LS mean change [90% CI] of 1.79 [1.36, 2.23] kg and 1.97 [1.57, 2.37] kg, respectively, vs. 0.64 [0.10, 1.18] kg for placebo). Accordingly, there were increases in percent body fat from baseline to day 22 in all treatment groups, with larger increases for olanzapine (1.68% [1.17, 2.20]) and OLZ/SAM (1.89% [1.42, 2.36]) compared with placebo (0.82% [0.18, 1.46]). Fat-free mass increased during treatment with olanzapine (1.13 [0.52, 1.74] kg) and OLZ/SAM (0.87 [0.31, 1.43] kg), but mean changes in fat-free mass with placebo were minimal (−0.06 [−0.81, 0.70] kg).

#### Caloric intake and energy expenditure

Caloric intake was measured at two single buffet-style meals: one at baseline and one at the end of the 3-week treatment period. At baseline, the mean (SD) total caloric intake was 1894.6 (488.2) kcal for placebo, 2198.3 (605.3) kcal for olanzapine, and 2374.0 (804.0) kcal for OLZ/SAM. Caloric intake increased in the placebo and olanzapine groups at day 22 (LS mean change [90% CI] of 92.6 [−188.98, 374.22] kcal and 201.6 [−10.90, 414.15] kcal, respectively), with the increase being more pronounced for olanzapine; Supplementary Fig. S5). In contrast, subjects in the OLZ/SAM treatment group consumed fewer calories at day 22 (LS mean change [90% CI] of −297.6 [−495.64, −99.55] kcal).

There were small mean increases in resting energy expenditure in all treatment groups. The greatest increase was observed for olanzapine (LS mean change [90% CI] of 67.9 [−13.64, 149.38] kcal/day), whereas changes for OLZ/SAM and placebo were similar (19.4 [−57.51, 96.38] kcal/day and 20.0 [−81.14, 121.24 kcal/day], respectively). Patients treated with olanzapine had an increased respiratory quotient over the 3-week treatment period (LS mean change from baseline to day 22 in ratio of CO_2_ eliminated/O_2_ consumed [90% CI]: 0.025 [0.01, 0.04]), which was larger than the changes from baseline with placebo (−0.002 [−0.03, 0.02]) or OLZ/SAM (0.003 [−0.02, 0.02]).

Carbohydrate oxidation increased from baseline to day 22 with olanzapine (LS mean change [90% CI]: 30.1 [4.97, 55.24] g/day), whereas placebo and OLZ/SAM were associated with smaller changes (−3.1 [−34.27, 28.02] g/day and 2.0 [−22.06, 26.11] g/day, respectively). Treatment with olanzapine resulted in decreased fat oxidation (LS mean change [90% CI]: −11.3 [−21.89, −0.79] g/day), whereas treatment with placebo or OLZ/SAM resulted in numeric increases (6.0 [−6.87, 18.96] g/day and 1.2 [−8.95, 11.27] g/day, respectively). Changes in protein oxidation were observed in all groups; however, there was a numeric decrease for placebo (LS mean change, −9.6 [−22.12, 3.01] g/day), whereas there were increases for olanzapine and OLZ/SAM (5.9 [−4.36, 6.18] g/day and 2.8 [−7.00, 12.63] g/day, respectively).

### Safety

In total, 49 of 60 subjects (81.7%) experienced at least 1 AE, with 9 (75.0%), 19 (79.2%), and 21 (87.5%) subjects reporting at least 1 AE during treatment with placebo, olanzapine, and OLZ/SAM, respectively (Table 2). Most AEs were mild (45 subjects; 75.0%) or moderate (3 subjects; 5.0%); only 1 (1.7%) subject experienced an AE of severe intensity (muscle spasm with olanzapine). In the six subjects who discontinued because of an AE, all AEs were mild or moderate in severity. AEs led to study discontinuation in two subjects on olanzapine (increased alanine aminotransferase in both) and in four subjects (six events) treated with OLZ/SAM (increased alanine aminotransferase [two subjects], increased aspartate aminotransferase [two subjects], dizziness [one subject], and fatigue [one subject]). No deaths or serious AEs were reported. AEs reported in at least five subjects in any treatment group were increased alanine aminotransferase, fatigue, somnolence, dry mouth, constipation, headache, increased aspartate aminotransferase, and dizziness (Table 2).

**Table 2:** Summary of adverse events.

Parameter	Placebo (*n* = 12)	Olanzapine (*n* = 24)	OLZ/SAM (*n* = 24)
Subjects with any AE, *n* (%)	9 (75.0)	19 (79.2)	21 (87.5)
AEs occurring in ≥10% of subjects,^a^ *n* (%)			
Increased alanine aminotransferase	0	11 (45.8)	6 (25.0)
Fatigue	1 (8.3)	7 (29.2)	7 (29.2)
Somnolence	0	2 (8.3)	7 (29.2)
Constipation	1 (8.3)	0	7 (29.2)
Dry mouth	0	0	8 (33.3)
Headache	2 (16.7)	1 (4.2)	5 (20.8)
Dizziness	2 (16.7)	0	5 (20.8)
Increased aspartate aminotransferase	0	2 (8.3)	5 (20.8)

*AE* adverse event, *OLZ/SAM* combination of olanzapine and samidorphan.

^a^In the overall safety population.

Clinically meaningful changes in weight (≥7% increase from baseline) occurred in 2 (16.7%), 9 (37.5%), and 6 (27.3%) subjects treated with placebo, olanzapine, and OLZ/SAM, respectively. Mean changes in blood pressure, heart rate, respiratory rate, and temperature were not considered clinically meaningful; in addition, no clinically meaningful changes occurred in hematology or ECG results. No subjects reported suicidal ideation or behavior, as assessed by changes in C-SSRS scores.

## Discussion

This short-term exploratory study replicated findings from previous studies in healthy volunteers in which olanzapine treatment induced early impairments in insulin sensitivity and lipid parameters that preceded weight gain [29–31]. The present study also identified early differences in the metabolic effects of olanzapine and OLZ/SAM that preceded the timing of divergences in weight trajectories that were previously reported [19, 23]. The insulin and C-peptide responses during an OGTT and changes in the insulinogenic index suggest a heightened insulin secretory response with olanzapine treatment but not with OLZ/SAM. As the AUC_Glu_ was similar between groups at baseline and there were similar small changes in both groups at day 19, this finding suggests a loss of insulin sensitivity associated with olanzapine treatment that did not occur to the same extent with OLZ/SAM. This finding is more clearly appreciated with the Matsuda index, a surrogate marker of insulin sensitivity that is calculated from glucose and insulin responses. Compared with placebo, olanzapine was associated with a marked reduction in the Matsuda index at day 19, whereas decreases were attenuated with OLZ/SAM.

Although no significant differences were observed between groups, the results of the MMTT were consistent with those of the OGTT. Similar changes in post-challenge glucose levels were observed in all treatment groups, but marked post-challenge hyperinsulinemia was observed with olanzapine, which superseded that observed with OLZ/SAM or placebo.

Olanzapine-induced hyperinsulinemia has been observed previously in studies of patients with schizophrenia [32, 33] and healthy volunteers [30, 34]. The findings of this study are consistent with reports that hyperinsulinemia results from increases in insulin secretion from the pancreas. Multiple mechanisms may account for olanzapine-mediated increases in insulin secretion, including alterations in neuronal modulation of the pancreas and even direct effects of dopamine receptor antagonism on pancreatic β-cells [35–37]. One study of intravenous glucose tolerance tests in healthy volunteers treated with olanzapine demonstrated an essential role of sympathetic input via muscarinic receptors in mediating the olanzapine-induced hyperinsulinemic response to glucose challenge [34]. More recently, it has been reported that pancreatic α- and β-cells not only express D2-like dopamine receptors but also are capable of local catecholamine synthesis [36]. The evidence suggests that antipsychotic-induced blockade of dopamine receptors may release dopamine-mediated inhibition of insulin secretion, leading to an increase in glucose-stimulated insulin secretion [35, 36].

The mechanisms through which samidorphan may attenuate this glucose-stimulated hyperinsulinemia are not clear. Although olanzapine and samidorphan target different receptors and likely act through distinct signaling pathways, it is possible that both pathways could converge on the same physiologic processes (i.e., insulin secretion). A role for the opioid system in regulating glucose homeostasis and the insulin secretory response has been established for decades [38–41]. Based on early studies, morphine- and β-endorphin-induced hyperglycemia result from both central and peripheral effects on the pancreas-adrenal axis and the pancreas [42, 43]. The opioid antagonist naltrexone has been reported to prevent the glucose-stimulated hyperinsulinemia associated with polycystic ovary syndrome [44]. Endogenous opioid peptides and μ- and δ-opioid receptors have been identified in pancreatic islets of various species, and opioids appear to exert both stimulatory and inhibitory effects on insulin secretion, depending on the concentration [38, 39, 45, 46]. These effects may be mediated by direct signaling on the β-cell and by potentiation of somatostatin signaling within the pancreas that inhibits insulin secretion [38, 39, 45, 46]. Taken together, there is an established role for opioidergic regulation of glycemic response at multiple levels (central and peripheral); therefore, it is feasible that samidorphan works independently, through opioid-related mechanisms, to oppose olanzapine’s hyperinsulinemic effects.

The higher the rates of endogenous glucose production and the higher the levels of fasting plasma insulin concentrations, the greater the HIR (and, typically, the more impaired is the hepatic insulin sensitivity) [47, 48]. Similar to the decreased insulin sensitivity detected with the Matsuda index over 3 weeks, olanzapine treatment also decreased hepatic insulin sensitivity, as assessed by the HIR index, whereas OLZ/SAM did not to the same degree. These findings suggest that the addition of samidorphan to olanzapine as OLZ/SAM may attenuate the negative effects of olanzapine on hepatic insulin sensitivity.

The findings on the Matsuda index and HIR index that samidorphan attenuated the effects of olanzapine on insulin sensitivity at day 21 are in contrast with data from the hyperinsulinemic-euglycemic clamp. For example, by clamp, both olanzapine and OLZ/SAM decreased insulin sensitivity with 10 days of treatment. However, by day 21, the changes in insulin sensitivity in both the olanzapine and OLZ/SAM groups were attenuated, with 90% CIs for the mean percent change from baseline in either active treatment group overlapping with those in the placebo group. The reason for this incongruence is unknown, but it is worth noting that the methodologies employed in this study assess glucose metabolism under different conditions.

As suggested by its name, the hyperinsulinemic-euglycemic clamp artificially establishes a state of constant hyperinsulinemia and euglycemia to measure how effectively insulin affects glucose uptake from blood. This experimental paradigm does not take into account other endogenous mechanisms that regulate glucose homeostasis under physiologic conditions, including changes in counter-regulatory hormones and neural endocrine responses to a meal. In contrast, the experimental conditions of the OGTT and MMTT reflect the body’s integrated response to a glucose challenge originating from the gastrointestinal tract, which involves more complex glucoregulatory changes, including gut hormone secretion, changes in glucagon secretion, and even glucose effectiveness [49] (i.e., the ability of glucose itself to suppress hepatic glucose production). The incongruence in results from the OGTT and clamp, therefore, suggests the possibility that OLZ/SAM and olanzapine differ primarily in how they affect those other endogenous mechanisms regulating glucose homeostasis, rather than in how they affect insulin signaling itself. A limitation of the current study is that aspects of glucose regulation, such as glucagon secretion and glucose effectiveness [49], were not measured.

This possibility is further supported by the similarity of results between the OGTT and MMTT. Although no statistical separation was observed, in both tests the elevated insulinogenic response with olanzapine was blunted with OLZ/SAM. Further research is needed to understand the precise mechanisms through which OLZ/SAM and olanzapine differentially regulate glucose homeostasis under physiologic conditions.

Although not the main focus of this study, caloric intake increased with olanzapine but decreased with OLZ/SAM. Both groups gained similar amounts of weight, but the opposing changes in caloric intake at endpoint may underlie the differential weight gain observed in longer studies, in which weight gain is similar for the first 4 to 6 weeks and then diverges [19, 21]. Olanzapine was also associated with an increased respiratory quotient and numerically greater resting energy expenditure not observed with OLZ/SAM or placebo, consistent with a previous study in healthy volunteers [50].

The findings from this study should be interpreted considering the following limitations. First, this study was exploratory in nature, with no predefined hypotheses and no correction for the multiple comparisons made. Second, this study was conducted in a relatively small number of healthy subjects who may have different tolerability to antipsychotic drugs than other patient populations [51]. As the study enrolled healthy volunteers, the dose of olanzapine could not exceed 10 mg, which is likely lower than doses administered to patients [52] (e.g., large population studies reported a median dose of 11.7 mg in outpatients [53] and mean doses of 18–19 mg among inpatients [54]). As such, the relevance of these data to patients with schizophrenia or bipolar disorder requires further study, and future research on the metabolic effects and mechanism of action of OLZ/SAM in patient populations should take these considerations into account. Finally, the adjustments to the regression model may not have fully corrected for the postrandomization imbalance in race between the treatment groups. This study was strengthened by the fact that it was metabolically controlled. The inclusion of healthy subjects who were free of underlying metabolic issues that might confound assessments also fortifies these findings [2, 24, 25].

Overall, OLZ/SAM was generally well tolerated, with an AE profile similar to that observed in a previous study of OLZ/SAM in healthy volunteers [55]. In this study, olanzapine and OLZ/SAM elicited early metabolic changes that preceded substantial or prolonged weight gain. Moreover, although the two treatments resulted in similar effects on lipid metabolism, they exhibited different effects on glucose metabolism and insulin sensitivity. For example, treatment with OLZ/SAM appeared to mitigate olanzapine-induced hyperinsulinemia and decline in the Matsuda index with OGTT and normalized the initial rise in the HIR index, findings that mirror early metabolic changes observed in recent nonclinical studies conducted in rodents and nonhuman primates [22]. These differences occurred when the weight gain profiles were similar and suggest differences in drug effects that are independent of weight. Although samidorphan has a pharmacodynamic profile distinct from that of olanzapine, they both may modulate relevant physiologic processes, including glucose and lipid metabolism.

## Supplementary information

SUPPLEMENTAL MATERIALS
