# Supplementary material for: Insulin and glucose metabolism with olanzapine and a combination of olanzapine and samidorphan: exploratory phase 1 results in healthy volunteers
Source: Neuropsychopharmacology. 2021 Dec 9;47(3):696–703. doi: 10.1038/s41386-021-01244-7 (PMC8782841; doi:10.1038/s41386-021-01244-7)

SUPPLEMENTAL MATERIALS

Methods

Results

Table S1. Treatment Difference of Fasting Glucose, Fasting Insulin, Homeostatic Model of Insulin Resistance, and Fasting Lipids

Table S2: Clamp-Derived Insulin Sensitivity Index Values Corrected for Fat-Free Mass at Baseline and at Days 10 and 21

Figure S1. Schematic of Stable Isotope-Labeled Glucose Infusions and 2-Step Hyperinsulinemic-Euglycemic Clamp Procedure

Figure S2. Patient Flow Diagram

Figure S3. Area Under the Plasma Concentration versus Time Curves for Analytes Measured During Tolerance Tests

Figure S4. HIR Index Corrected for Fat-Free Mass at Day 21

Figure S5. Change From Baseline to Day 22 in Caloric Intake at a Single Meal

METHODS

Assessments

Metabolic Tolerance Tests (OGTT and MMTT)

Glucose metabolism was assessed during an oral glucose tolerance test (OGTT). Immediately after a blood draw at the 0-hour time point, subjects were given an oral glucose solution (75 g) to consume within a 5-minute period. Additional blood samples were taken over the next 180 minutes. Absolute and incremental changes in glucose, insulin, and C-peptide, the area under the plasma concentration-time curve from 0 to 180 minutes ($AUC_{0\text{–}180\ min}$), and the Matsuda index (2-hour) were calculated. The Matsuda index, an indicator of insulin sensitivity, was derived from fasting values of insulin and glucose and mean values during the OGTT, per the following formula:

$$\frac{10000}{\sqrt{\text{Glucose}_0[\text{mg/dL}]*\text{Insulin}_0[\mu\text{U/mL}]*\left(\frac{\text{AUC}_{\text{Gluc }0\text{–}120\text{ min}}}{120\text{ min}}\right)*\left(\frac{\text{AUC}_{\text{Ins }0\text{–}120\text{ min}}}{120\text{ min}}\right)}}$$

For the mixed-meal tolerance test (MMTT), subjects consumed two 8-ounce bottles of Ensure Plus, each of which contained 350 kcal and standard MMTT ratios of fat, carbohydrate, and protein. Blood samples were collected for 360 minutes post-supplement administration for calculation of absolute and incremental changes in glucose, insulin, and area under the plasma concentration-time curve from 0 to 360 minutes ($AUC_{0\text{–}360\ min}$).

Endogenous Glucose Production and Hyperinsulinemic-Euglycemic Clamp

Subjects received a standardized dinner, followed by a 10-hour fast. Whole-body insulin sensitivity was assayed by a combination of stable isotope-labeled glucose infusion (to

assess endogenous glucose production) and 2-step hyperinsulinemic-euglycemic clamp, which infused insulin first at a low (step 1), then a high rate (step 2).

Infusions of sterile 20% dextrose (D-glucose) solution in water ($D_{20}W$) and insulin were administered by forearm cannula; blood glucose concentrations and insulin samples were taken from the contralateral side. The exogenous dextrose solution was enriched with [6,6-$^2H_2$]glucose to achieve isotope enrichment comparable to that of plasma in the fasting state (eg, 2.5% to 3% for [6,6-$^2H_2$]glucose).

The glucose enrichment in the blood was measured using gas chromatography–mass spectrometry, and EGP was calculated from the dilution of the infused stable isotope–labeled glucose using the steady-state method described by Steele [2]: EGP= $\frac{\text{rate of glucose tracer infusion}}{\text{steady-state plasma glucose enrichments}}$ – total glucose infusion rate.

During the 2-step hyperinsulinemic-euglycemic clamp procedure, a primed continuous infusion of [6,6-$^2H_2$]glucose and the hot glucose infusate (GINF) approach (**Supplementary Figure S1**) was used [1]. First, subjects were connected to a glucose analyzer for at least 2 hours, which automatically calculated the appropriate glucose infusion rate to keep blood glucose at the target level (90 mg/dL). Second, 5 mg of [6,6-$^2H_2$]glucose/kg body weight was injected over 3 minutes, followed by a constant infusion of 0.05 mg [6,6-$^2H_2$]glucose/kg body weight/min (initiated prior to and continued during the clamp). Then, the rate of the labeled glucose infusion was reduced by 50% coinciding with the start of the clamp. A low dose of insulin (Humulin R U-100; Eli Lilly

and Co., Indianapolis, IN, USA) was infused intravenously by means of a precision pump at a rate of 12 mU/m^2/min for 180 minutes. This was followed by a high-dose insulin infusion of 40 mU/m^2/min for 180 minutes. Subjects remained fasting (although they could sip water) and in a supine or semi-supine position during the entire clamp procedure.

Glucose disposal rates for each step were expressed as M, defined as the glucose infusion rate per minute, or R_d, which is based on stable isotope-labeled glucose tracer dilution method; both were corrected for fat-free mass. The insulin sensitivity index was calculated as follows: $\frac{\text{mean glucose infusion rate}_{\text{Step2}} - \text{mean glucose infusion rate}_{\text{Step1}}}{[\text{mean insulin}_{\text{Step2}} - \text{mean insulin}_{\text{Step 1}}] * [\text{mean blood glucose}_{\text{Steps1\&2}}]}$.

Hepatic Insulin Resistance Index

The HIR index (measured before initiation of the clamp procedure), is an indicator of the ability of insulin to suppress endogenous hepatic glucose production in the fasting state. The HIR was calculated in an additional analysis (ie, not prespecified) as change from baseline to day 21 in fasting endogenous glucose production × fasting insulin values.

Energy Intake/Expenditure and Substrate Oxidation

Indirect calorimetry was performed to assess resting metabolic rate and substrate oxidation. No physical activity (with the exception of a short visit to the lavatory and registration of body weight) or food or liquid intake were allowed before this assessment; subjects rested in a comfortable bed for 30 minutes prior to and during these measurements. A ventilated hood was placed over the head of the subject for

continuous registration of resting metabolic rate (ie, resting energy expenditure), substrate oxidation, and respiratory quotient. Total caloric intake was based on the amount of food consumed during a buffet-style meal (developed by a dietician) given prior to treatment and again after the last day of treatment; change from baseline was calculated.

Additional Assessments

Body composition, including fat mass, fat-free mass, and percentage body fat, was assessed using dual energy x-ray absorptiometry (DEXA). Safety and tolerability were evaluated by the incidence of adverse events (AEs), including serious AEs and AEs leading to discontinuation, and by monitoring vital signs, laboratory findings, electrocardiograms, and Columbia-Suicide Severity Rating Scale (C-SSRS) scores over the course of the study.

RESULTS

Supplementary Table S1 summarizes fasting laboratory values at baseline and changes from baseline that occurred over the 3-week treatment period.

Mean values at baseline and the absolute change and percentage change from baseline at days 10 and 21 for the composite Si measure are depicted in **Supplementary Table S2.**

The plasma concentration profiles over time at baseline and endpoint for glucose,

insulin, and C-peptide during tolerance tests are presented in **Supplementary Figure S2**.

The ability of insulin to suppress endogenous glucose production in the fasting state was assessed by the HIR index at baseline, day 10, and day 21 (**Supplementary Figure S3**).

Supplementary Figure S4 depicts the effects of placebo, olanzapine, and OLZ/SAM treatment on caloric consumption during a buffet-style meal after 3 weeks of treatment relative to caloric intake at a buffet-style meal given prior to administration of study drug.

REFERENCES

1. Finegood DT, Bergman RN, Vranic M. Estimation of endogenous glucose production during hyperinsulinemic-euglycemic glucose clamps. Comparison of unlabeled and labeled exogenous glucose infusates. *Diabetes.* 1987;36(8):914-924.

2. Steele R. Influences of glucose loading and of injected insulin on hepatic glucose output. Ann N Y Acad Sci. 1959;82:420-430.

TABLES

Table S1. Treatment Difference of Fasting Glucose, Fasting Insulin, Homeostatic Model of Insulin Resistance, and Fasting Lipids

Parameter	Placebo (n=12)	Olanzapine (n=24)	OLZ/SAM (n=24)
Fasting glucose			
Baseline, mean (SD), mg/dL	93.7 (6.5)	95.9 (5.0)	94.0 (6.5)
Change from baseline to day 22, LS mean (SE), mg/dL[a,b]	−8.4 (1.9)	−7.5 (1.6)	−5.0 (1.4)
90% CI	−11.63, −5.14	−10.06, −4.85	−7.33, −2.75
Fasting insulin			
Baseline, mean (SD), mg/dL	6.2 (3.1)	8.0 (4.6)	5.8 (2.7)
Change from baseline to day 22, LS mean (SE), mg/dL[b,c]	−0.9 (1.4)	1.2 (1.1)	1.2 (0.9)
90% CI	−3.18, 1.48	−0.60, 3.04	−0.43, 2.74
Homeostatic model of insulin resistance			
Baseline, mean (SD), mg/dL	1.45 (0.78)	1.90 (1.13)	1.36 (0.65)
Change from baseline to day 22, LS mean (SE), mg/dL[c]	–0.35 (0.36)	0.22 (0.25)	0.11 (0.28)
90% CI	–0.96, 0.26	–0.20, 0.63	–0.37, 0.58
Fasting free fatty acid			
Baseline, mean (SD), mmol/L	0.38 (0.19)	0.43 (0.18)	0.50 (0.24)
Change from baseline to day 22, LS mean (SE), mmol/L[a,b]	−0.12 (0.05)	−0.14 (0.04)	−0.11 (0.03)
90% CI	−0.20, -0.04	−0.21, -0.08	−0.16, -0.05
Fasting cholesterol			
Baseline, mean (SD), mg/dL	162.2 (26.3)	163.2 (30.5)	155.0 (26.4)
Change from baseline to day 22, LS mean (SE), mg/dL[b–d]	−4.8 (7.2)	13.9 (5.6)	18.9 (4.9)
90% CI	−16.91, 7.35	4.59, 23.20	10.71, 27.16
Fasting HDL cholesterol			
Baseline, mean (SD), mg/dL	53.4 (7.5)	54.9 (14.5)	58.2 (11.9)
Change from baseline to day 22, LS mean (SE), mg/dL[b,c]	−7.1 (2.8)	−9.4 (2.1)	−5.4 (1.9)
90% CI	−11.69, −2.44	−13.00, −5.88	−8.47, −2.24
Fasting LDL cholesterol			

Baseline, mean (SD), mg/dL	100.6 (24.9)	98.6 (26.4)	90.1 (25.0)
Change from baseline to day 22, LS mean (SE), mg/dL[a,e]	2.3 (5.8)	20.4 (4.7)	23.7 (4.2)
90% CI	−7.48, 12.05	12.60, 28.22	16.76, 30.70
Fasting triglycerides			
Baseline, mean (SD), mg/dL	83.7 (34.4)	87.2 (44.0)	69.6 (27.7)
Change from baseline to day 22, LS mean (SE), mg/dL[a,b]	1.0 (8.7)	11.3 (7.0)	2.0 (6.2)
90% CI	−13.50, 15.46	−0.39, 22.90	−8.39, 12.32

$AUC_{0\text{-}22h}$, area under the plasma concentration-time curve from time 0 to 22 hours; HDL, high-density lipoprotein; LDL, low-density lipoprotein; LS, least squares; OLZ/SAM, combination of olanzapine and samidorphan.

[a]Subjects with available data at day 22 were n=22, n=24, and n=11 for OLZ/SAM, olanzapine, and placebo groups, respectively.

[b]There were no differences in change from baseline to day 22 values between active treatment groups and placebo for fasting glucose, insulin, free fatty acids, HDL cholesterol, and triglycerides.

[c]Subjects with available data at day 22 were n=22, n=24, and n=10 for OLZ/SAM, olanzapine, and placebo groups, respectively.

[d]The LS mean difference (90% CI) between olanzapine and placebo at day 22 versus baseline for total cholesterol was 18.7 (3.84–33.51) mg/dL; the LS mean difference between OLZ/SAM and placebo was 23.7 (8.92–38.51) mg/dL.

[e]The LS mean difference (90% CI) between olanzapine and placebo at day 22 versus baseline for LDL cholesterol was 18.1 (5.79–30.46)] mg/dL; the LS mean difference between OLZ/SAM and placebo was 21.4 (9.32–33.56) mg/dL) .

Table S2. Clamp-Derived Insulin Sensitivity Index Values Corrected for Fat-Free Mass at Baseline and at Days 10 and 21

Parameter	Placebo	Olanzapine	OLZ/SAM
Baseline			
n	12	24	24
Mean (SD), mL*mL/min/uIU/kg	0.21 (0.09)	0.24 (0.11)	0.22 (0.06)
Day 10			
n	12	24	22
LS mean (SE) change from baseline, mL*mL/min/uIU/kg	−0.04 (0.01)	−0.10 (0.01)	−0.09 (0.01)
Mean percent (90% CI) change from baseline, %	−9.2 (−21.8, 3.3)	−37.9 (−48.3, −27.4)	−36.5 (−45.8, −27.3)
Day 21			
n	11	23	19
LS mean (SE) change from baseline, mL*mL/min/uIU/kg	−0.04 (0.02)	−0.06 (0.01)	−0.06 (0.01)
Mean percent (90% CI) change from baseline, %	−6.1 (−19.3, 7.1)	−21.5 (−32.9, −10.1)	−26.6 (−36.6, −16.6)

LS, least squares; OLZ/SAM, combination of olanzapine and samidorphan.

FIGURES

Figure S1. Schematic of Stable Isotope-Labeled Glucose Infusions and Two-Step Hyperinsulinemic-Euglycemic Clamp Procedure. Infusion of tracer glucose (6,6-D_2-glucose) at a rate of 0.05 mg/kg body weight/min over 6 hours was initiated prior to the start of the clamp to allow for equilibration. Blood samples (indicated by red arrows) were taken before the start of the glucose infusion and before the start of and during the 2-step clamp. Basal endogenous glucose production (EGP) was assessed based on measurements taken in the 60 minutes prior to initiation of the clamp.

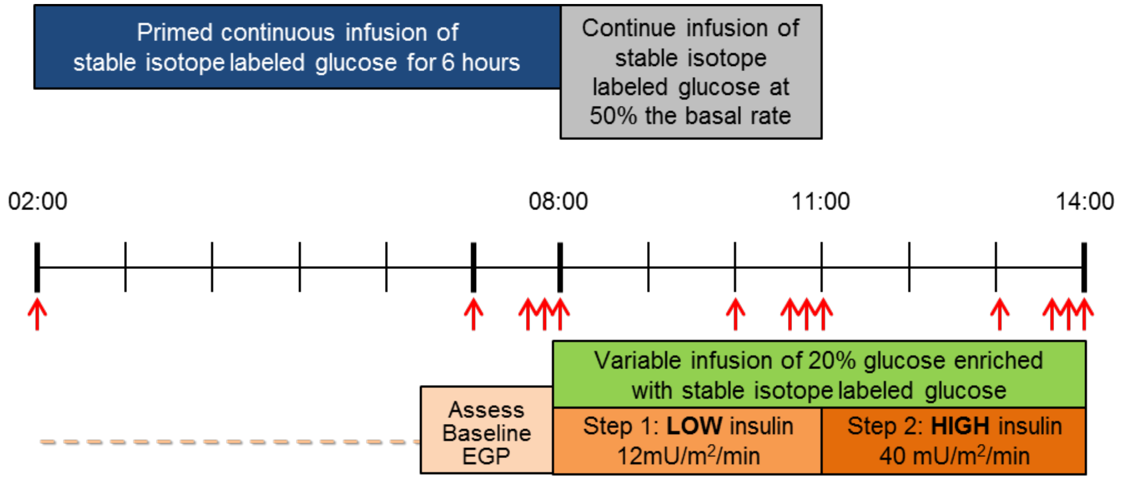

Figure S2. Patient Flow Diagram

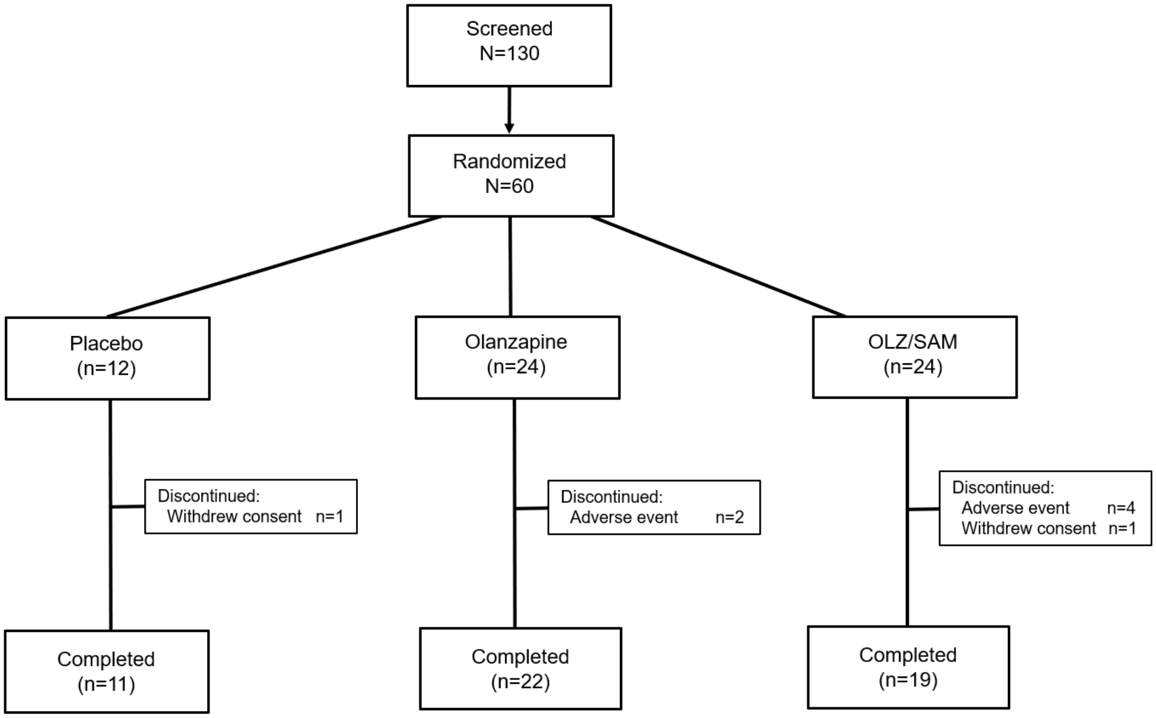

Figure S3. Area Under the Plasma Concentration Versus Time Curves for Analytes Measured During Tolerance Tests. The effects of placebo, olanzapine, or OLZ/SAM on postprandial glucose-related parameters were assessed after oral administration of 75 g of glucose for 180 minutes (OGTT) or two 8-ounce bottles of Ensure Plus for 360 minutes (MMTT). Concentrations versus time profiles for glucose, insulin, and C-peptide values during the OGTT at baseline and day 19 are presented in the upper, middle, and lower sections, respectively, of Figure S2A by treatment group. Concentration versus time profiles at baseline and at day 18 of glucose, insulin, and C-peptide profiles during the MMTT are depicted in Figure S2B (upper, middle, and lower sections, respectively) by treatment group.

MMTT, mixed meal tolerance test; OGTT, oral glucose tolerance test; OLZ/SAM, combination of olanzapine and samidorphan.

A. OGTT

Glucose

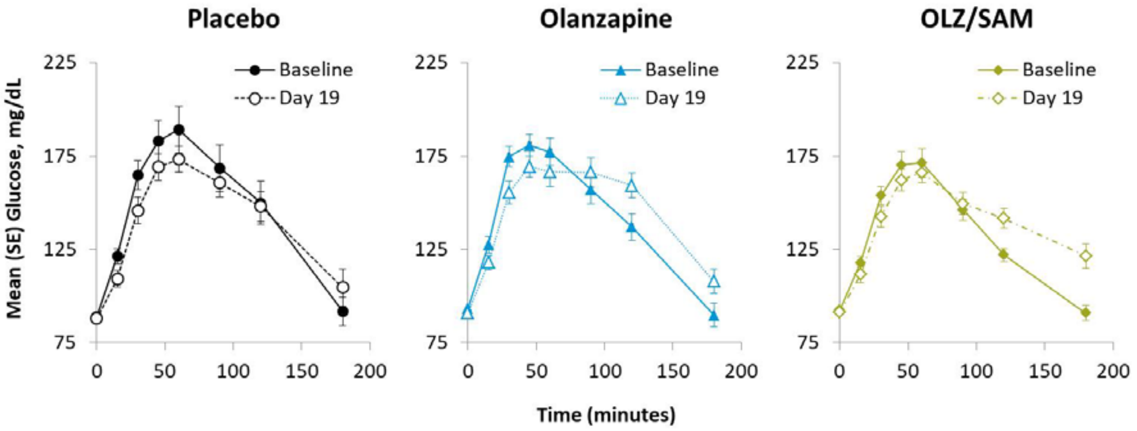

Insulin

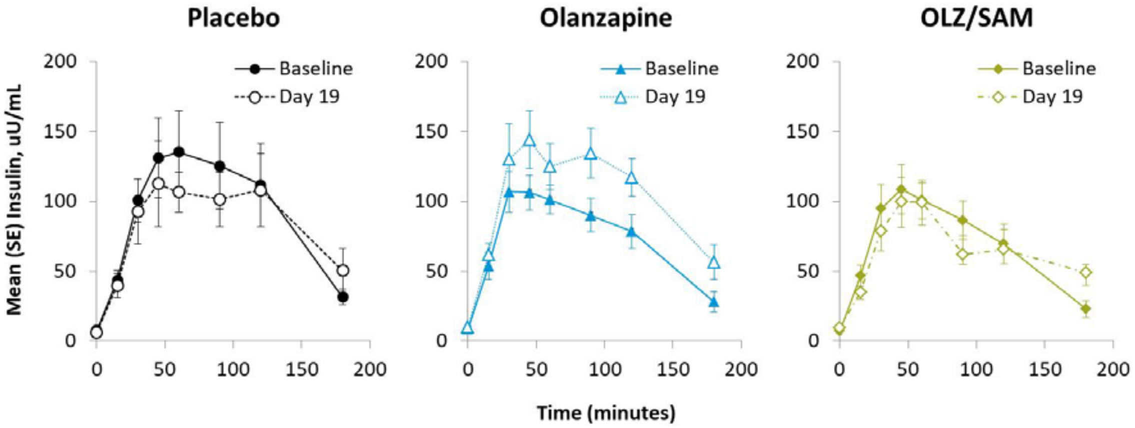

C-peptide

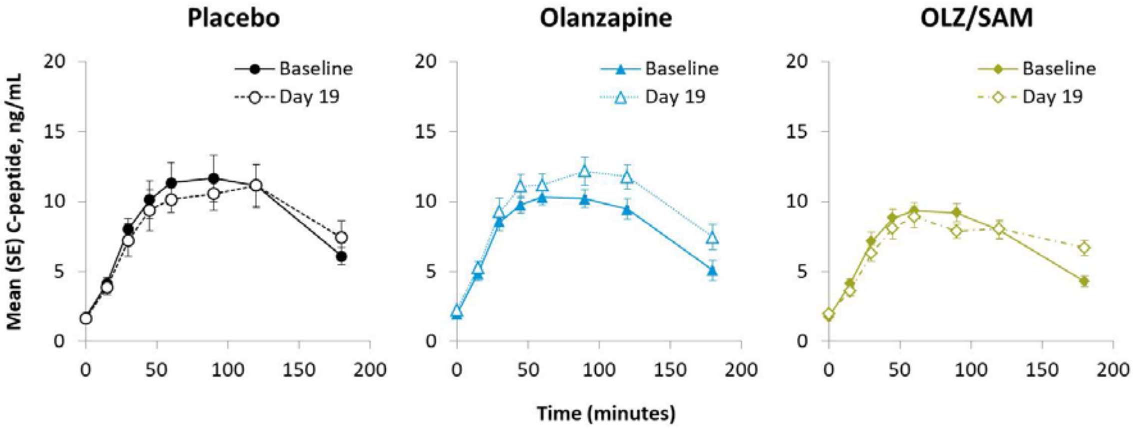

B. MMTT

Glucose

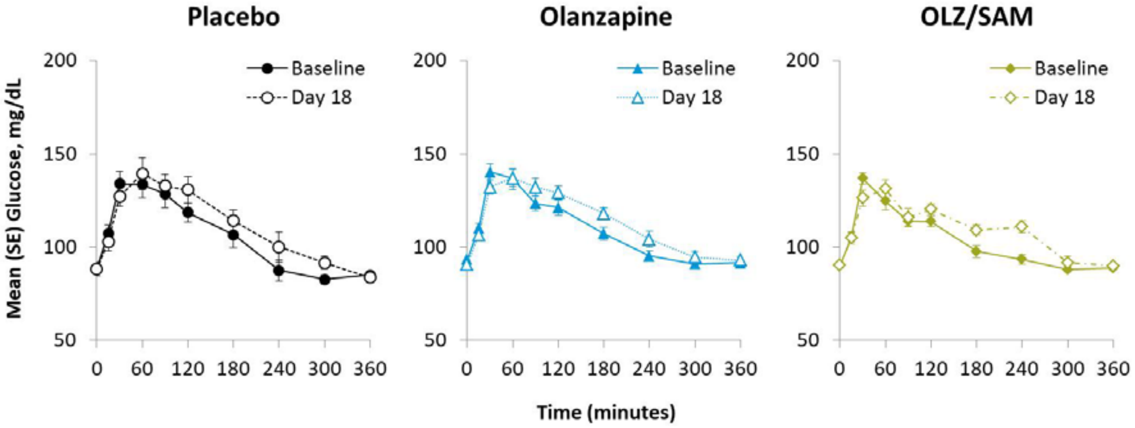

Insulin

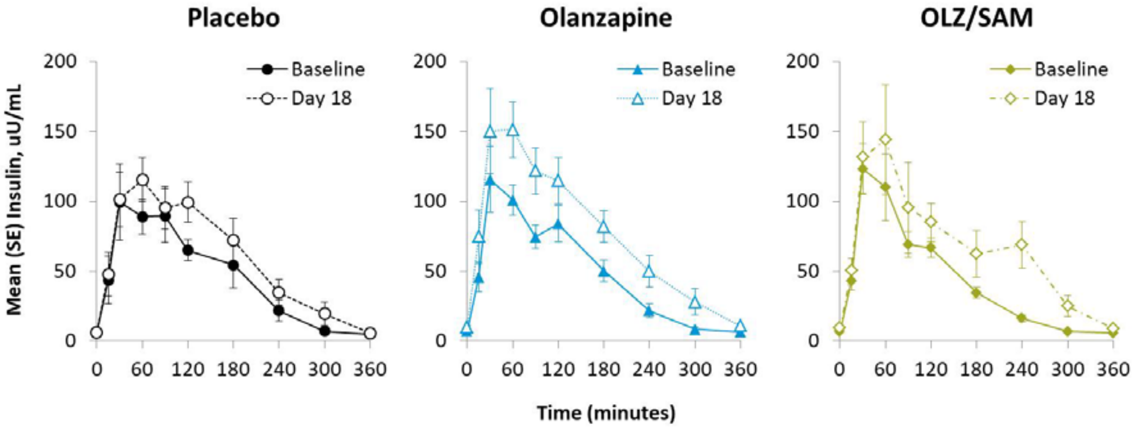

C-peptide

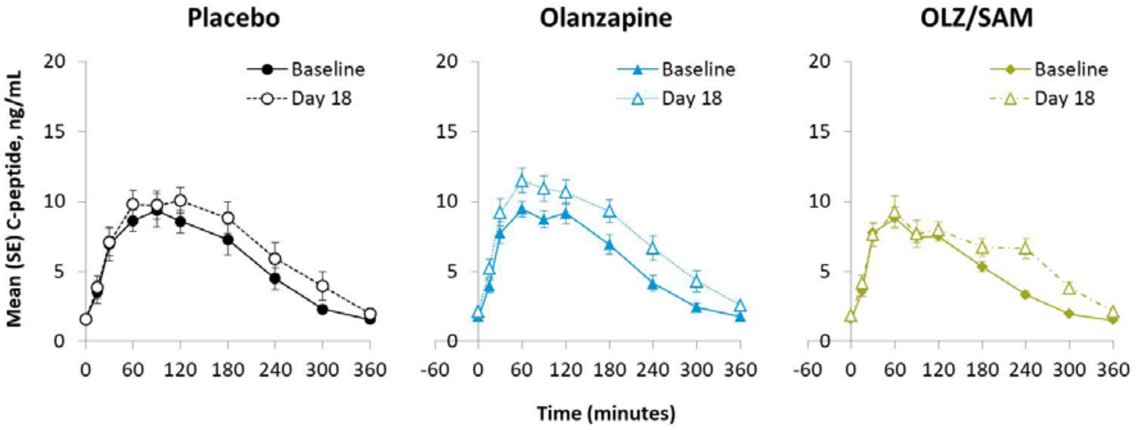

Figure S4. HIR Index Corrected for Fat-Free Mass at Day 21. The LS mean was generated using ANCOVA, with treatment as a factor and baseline of the dependent variable and race (black/African American or not) as covariates.

ANCOVA, analysis of covariance; FFM, fat-free mass; HIR, hepatic insulin resistance; LS, least squares; OLZ/SAM, combination of olanzapine and samidorphan.

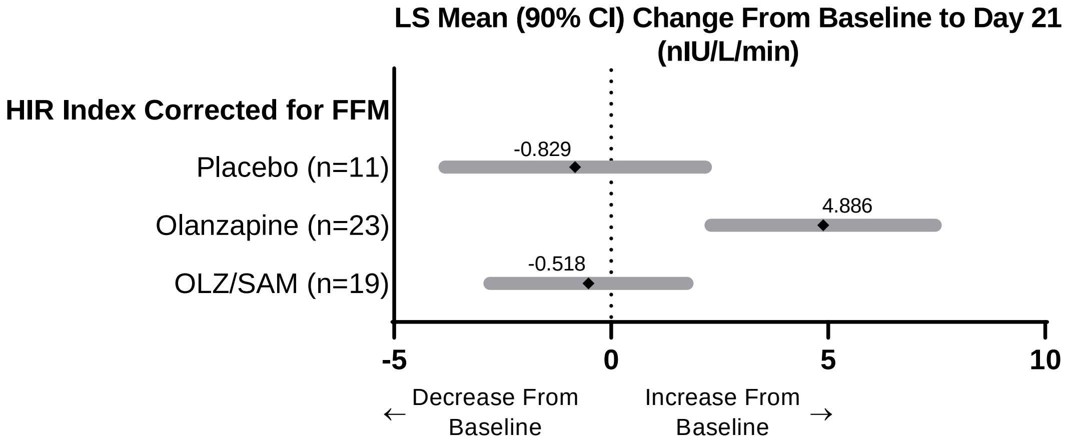

Figure S5. Change From Baseline to Day 22 in Caloric Intake at a Single Meal.

Subjects were given a buffet-style meal on days 2 and day 22, at which time, food intake was measured by study personnel. LS means were generated using ANCOVA, with treatment as a factor and baseline for the dependent variable and race (black or not) as covariates.

ANCOVA, analysis of covariance; LS, least squares; OLZ/SAM, combination of olanzapine and samidorphan.

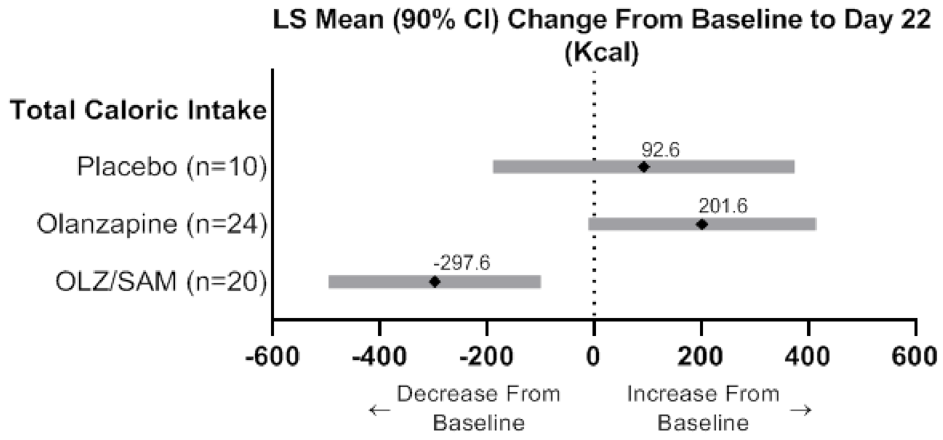

Supplement: Supplementary file 1 — SUPPLEMENTAL MATERIALS [file 41386_2021_1244_MOESM1_ESM.pdf]
